# Tryptamine-Functionalized Lipid Nanocarriers Co-delivering SMO/BRD4 Inhibitors for Synergistic Medulloblastoma Therapy

**DOI:** 10.34133/bmr.0237

**Published:** 2025-08-08

**Authors:** Qiyue Wang, Zixu Cui, Chenguang Guo, Yue Zhang, Jinhua Chen, Ruitao Zhang, Xueming Li, Zhengjie Meng, Hao Ren

**Affiliations:** ^1^ School of Pharmaceutical Science, Nanjing Tech University, Nanjing 211816, China.; ^2^ NMPA Key Laboratory for Research and Evaluation of Pharmaceutical Preparations and Excipients, China Pharmaceutical University, Nanjing 210009, China.; ^3^ Department of Pharmaceutics, School of Pharmacy, China Pharmaceutical University, Nanjing 211198, China.; ^4^ College of Biotechnology and Pharmaceutical Engineering, Nanjing Tech University, Nanjing 211816, China.

## Abstract

The management of medulloblastoma (MB) remains a significant challenge, primarily attributed to the presence of cancer stem cells and the inadequate delivery of therapeutic agents across the blood–brain barrier. GLI, as a regulator of the hedgehog signaling pathway in normal cerebellum development, also exerts pivotal functions in MB initiation, progression, and metastasis and maintains the stemness of MB stem cells. In this study, we devised a combined therapeutic approach by integrating the BRD4 inhibitor JQ1 with the SMO inhibitor saikosaponin B1 (SSB1) to inhibit MB via regulation of GLI activation. The results suggested that JQ1 and SSB1 synergistically inhibited MB proliferation, constricted MB metastasis, and down-regulated stem cell phenotypes via reduced GLI and MYC expression. Tryptamine-derived lipid nanoparticles (NPs) transported JQ1 and SSB1 to MB tissues. The targeted NPs demonstrated prolonged drug release kinetics and significantly improved their accumulation in MB tumors. Systemic administration of drug-loaded targeted NPs significantly decreased tumor burden without hepatic toxicity in xenograft MB-bearing mice. The combination of JQ1 and SSB1 presents an innovative therapeutic paradigm for suppressing MB proliferation, recurrence, and metastasis, with the potential to drive the development of novel MB treatment strategies in the future.

## Introduction

Medulloblastoma (MB) is one of the most common malignant tumors in the cerebella of children [1]. Current MB treatment includes surgery and a high dose of radiation followed by chemotherapy [2]. Despite recent advances, over 30% of children die from MB, and 30% suffer from tumor recurrence [3–5]. Most survivors are left with endocrine disorders and lifelong neurocognitive sequelae because of the neurotoxicity associated with radiation and chemotherapeutic drugs. Therefore, more accurate and effective therapeutic strategies against MB with minimal toxicity are urgently needed to improve the patient’s survival.

Whole-genome sequencing and gene copy number analysis have enabled the classification of MB into 4 major subtypes: wingless/integrated, sonic hedgehog (SHH), group 3, and group 4 [6,7]. Almost 30% of all patients with MB belong to the SHH group characterized by the activation of the hedgehog (Hh) signaling pathway, and 25% belong to the group 3 subtype with *MYC* gene amplification. The activation of GLI1, 1 of 3 isoforms, is often considered a readout of Hh pathway activation, regulating genes such as *MYCN*, *CYCLIN D*, and *BCL2* to control tumor progression [8,9]. Also, in the group 3 subtype, MYC as a transcription factor could regulate GLI1 expression, leading to tumor growth and relapse. Therefore, regulation of GLI1 activity is an attractive strategy for MB treatment in SHH MB and group 3 MB.

It has been reported that the Hh pathway regulates cancer stem cell (CSC) proliferation [10,11]. CSCs are a small population of tumor cells with self-renewal and differentiation capacity to generate tumors. In recent years, CSCs have been found in different subtypes of MB [12–15]. The stemness property of CSCs in MB allows postsurgical relapse and drug resistance. Several critical regulators have been found to contribute to CSC maintenance. Transcription factors GLI1/2 have been reported as crucial effectors to regulate stemness-related genes such as *MYCN*, *OCT4*, *NANOG*, and *BMI1* [12–14,16]. The metastasis of MB is also related to CSCs through the regulation of the TWIST1/BMI1 axis [17–20]. BMI1 could promote the epithelial–mesenchymal transition process with loss of cell adhesion and induce MB invasion into healthy tissues. Moreover, MB metastasis is also facilitated by matrix metalloproteinases (MMPs), such as MMP2 and MMP9, by extracellular matrix (ECM) degradation, and later, MMPs are up-regulated by BRD4/MYC and GLI [21–23]. Meanwhile, for group 3 MB, MYC could directly regulate stemness-related factors and indirectly regulate *GLI* and its downstream genes.

Several strategies have been developed to eliminate CSCs, such as blocking antiapoptosis pathways, maintaining the quiescence of CSCs, or disrupting the interaction between CSCs and the microenvironment [24,25]. The most effective strategies focus on target-specific signaling pathways such as the NOTCH pathway, PI3K/AKT pathway, and SHH pathway to reduce CSC frequency [16,26,27]. However, inhibition of SHH MB is associated with the development of chemoresistance because of mutations of *SMO* and *SUFU* [28,29]. On the other hand, direct MYC inhibition has been proven to be a difficult therapeutic target, and inhibition of PI3K to enhance MYCN degradation provides only limited therapeutic benefits and leads to activation of compensatory pathways and chemoresistance [30]. These resistance mechanisms could be overcome by modulating *GLI* transcription downstream of *SMO* using JQ1, which is a BRD4 inhibitor and inhibits *MYC* expression and the proliferation of CD15^+^ cells (CSCs).

Efficient transport of drugs across the blood–brain barrier (BBB) is still a considerable challenge [31]. The BBB is a physiological barrier that regulates the entry of molecules into the brain by restricting their passive transportation [32,33]. With nanotechnology development, several delivery systems were established to improve the biodistribution of drugs in the brain [34]. Nanoplatforms have the advantages of a high loading capacity, a long circulation half-life, and reduced toxicity by providing controlled release [35,36]. Further, nanoformulations decorated by certain antibodies or protein fragments could directly access the brain through receptor-mediated endocytosis. Several targeting ligands for brain endocytosis have been reported, such as the insulin-like growth factor 1 receptor (IGF1R), transferrin receptor, and low-density lipoprotein receptor [37–41]. However, the widespread expression of these receptors in other primary tissues resulted in unintended biodistribution patterns and potential toxicity risks and limited the usage of brain-targeting delivery platforms. A targeting ligand specific to the desired organ/site could better control the distribution of nanoparticles (NPs). Some neurotransmitters, as endogenous chemicals, have been demonstrated to cross the BBB [42]. It has been reported that tryptamine (Try)-derived synthetic lipids effectively deliver drug-loaded cargo into the brain [43,44]. Even though the BBB transportation mechanism of neurotransmitter-derived carriers is still not fully understood, several investigations suggested that organic cation transporters (OCTs) might be the targeted receptors for neurotransmitters transporting into the brain. The rich expression of OCTs in the brain microvascular endothelial cells suggests their substrates, such as Try, as attractive ligands for decorated nanoplate forms targeted to the brain. Moreover, vigorous tumor metabolism facilitated the cellular uptake of nutrients, including Try, which could potentially improve MB targeting.

In this study, we developed a therapeutic strategy to co-deliver the BRD4 inhibitor JQ1 and the SMO inhibitor saikosaponin B1 (SSB1) (extracts from Chinese Thorowax roots) using tryptamine-derived lipid (Try-Lip) NPs for targeted MB treatment. Try-Lip NPs achieved satisfactory BBB penetration ability based on the organic cation transporter 2 (OCT2)-mediated assay. The combination of JQ1 and SSB1 efficiently reduced MB cell viability by regulating the cell cycle and apoptosis, significantly reduced CD15^+^ MB stem cell population, and down-regulated the expression of metastasis-related factors, exhibiting significant MB progression and metastasis inhibition ability. Systemic administration of Try-derived NPs significantly improved both JQ1 and SSB1 accumulation in orthotopic MB and efficiently decreased tumor burden in vivo. Therefore, combining a BRD4 inhibitor and an SMO inhibitor appears to be a very attractive therapeutic avenue for MB treatment.

## Materials and Methods

### Materials

SSB1, GDC-0449, and JQ1 were procured from MedChemExpress (Princeton, USA). Try-Lips were synthesized in-house. DSPE-PEG_2k_ and distearoylphosphatidylcholine (DSPC) were purchased from AVT Pharmaceutical Tech Co., Ltd. (Shanghai, China). All other chemicals and reagents were purchased from Sinopharm Chemical Reagent Co., Ltd. (Shanghai, China).

### Mammalian cells and animals

DAOY and D341 Med cells were procured from Wuhan Pricella Biotechnology Co., Ltd. (Wuhan, China). ONS-76 cells were procured from Shanghai Yu Bo Biotech Co., Ltd. (Shanghai, China). SVG p12 cells were procured from Xiamen Immocell Biotechnology Co., Ltd. (Fujian, China). Human brain microvascular endothelial cells (HBMECs) were procured from Hefei Wanwu Biotechnology Co., Ltd. (Anhui, China). DAOY and D341 Med cell lines were maintained in Eagle’s minimum essential medium supplemented with 10% fetal bovine serum (FBS) and a 1% penicillin/streptomycin/gentamicin B cocktail. ONS-76 and SVG p12 cells were cultured in Dulbecco’s modified Eagle’s medium (DMEM), containing 10% FBS and 1% penicillin/streptomycin/gentamicin B. HBMECs were cultured in MCDB 131 medium supplemented with 10 ng/ml epidermal growth factor, 1 μg/ml hydrocortisone, 10 mM glutamine, and 10% FBS. Cells were maintained in a humidified 37 °C incubator supplemented with 5% CO_2_ for further use. CD15^+^ DAOY and CD15^+^ ONS-76 cells were sorted from DAOY and ONS-76 cells using a flow cytometer after incubating with BV-650-CD15 antibody. The sorted cells were seeded onto a flask in DMEM/F12 media supplemented with 1 × N2, 0.5 × B27 without vitamin A, 10 ng/ml epidermal growth factor, and 10 ng/ml basic fibroblast growth factor and maintained at 37 °C in a humidified atmosphere with 5% CO_2_.

NSG mice at 6 to 8 weeks with random sex were procured from Shanghai Model Organisms Center, Inc. (Shanghai, China). All animal procedures were authorized by the Institutional Animal Care and Use Committee of the Animal Experiment Center at China Pharmaceutical University (Approval No. 20190515-007). All mice were kept in a specific-pathogen-free-grade facility under a 12-h light/dark cycle, at 20 ± 3 °C, and with 40% to 70% relative humidity.

### Synthesis of Try-Lips

Try-Lips were synthesized with a 4-step reaction, as shown in Fig. S1. (a) Ten grams of 2,2′-pyridine disulfide and 0.67 ml of acetic acid were mixed in methanol. A solution of undecanethiol (5.09 ml) in 15 ml of dichloromethane was added dropwise to the mixture, followed by stirring at room temperature for 3 h. The solvent in the mixture was evaporated, and compound A was purified using silicone column chromatography assay with ethyl acetate and hexane (1:9 *v*/*v* ratio) as the eluent. (b) Compound A (4.0 g) was dissolved in 50 ml of methanol with a catalytic amount of acetic acid, and 2-mercaptoethanol (2.1 g) was added dropwise, followed by a 6-h reaction at room temperature. The solvent of the mixture was evaporated, and compound B was purified using silicone column chromatography assay with a mixture of ethyl acetate and hexane (1:4 *v*/*v* ratio) as the eluent. (c) Compound B (1.3 g) was dissolved in 50 ml of dichloromethane and mixed with 1.0 ml of triethylamine. The resulting solution was cooled to 0 °C, and acryloyl chloride (0.56 g) was added dropwise. The reaction mixture was then stirred at room temperature for 4 h, followed by extraction with dichloromethane at least 3 times. The organic phases were merged and concentrated under reduced pressure. Compound C was purified using silicone column chromatography assay with a mixture of ethyl acetate and hexane (1:4 *v*/*v* ratio) as the eluent. (d) To synthesize Try-Lips, Try and compound C were mixed and heated at a 1:2.4 molar ratio at 80 °C for 48 h. The crude product was purified by silica gel column chromatography, and the structure of Try-Lip and intermediate products was characterized by ^1^H nuclear magnetic resonance.

### Preparation and characterization of NPs

Try-derived NPs were prepared using the film hydration assay. Briefly, Try-Lips, cholesterol, DSPC, and DSPE-PEG_2k_ at a molar ratio of 50:38.5:10:1.5 (total mass of 30 mg) were dissolved in chloroform and mixed with 1.5 mg of JQ1 and 3.0 mg of SSB1. The solvent was evaporated to form a film, followed by overnight drying in a vacuum. Then, 2 ml of PBS was added, and the mixture was sonicated in a 37 °C water bath for 10 min. The mixture was then extruded into a film with a pore size of 200 nm at least 5 times. The solution was collected for further characterization. The hydrodynamic diameter and zeta potential of the NPs were analyzed by Malvern Zetasizer. The particle morphology was evaluated using transmission electron microscopy. The drug loading (DL) and encapsulation efficiency of the formulation were measured using a high-performance liquid chromatography system (Waters Corp). The drug release behavior was analyzed by placing drug-loaded NPs (equivalent 500 μg) into a dialysis bag and suspended into 50 ml of PBS solution containing 1% *v*/*v* Tween 80. At specified time points, 1 ml of the release medium was collected, and an equal volume of fresh medium was replenished. The drug concentration in the samples was analyzed by high-performance liquid chromatography to determine the release profile. The stability of drug-loaded particles was determined by measuring the particle size at different time intervals after the formulation was prepared. The nontargeted NPs were prepared with the formulation of cholesterol, DSPC, and DSPE-PEG_2k_ using the same assay.

### Cell viability assay

Cells were plated into 96-well plates at a density of 3 × 10^3^/well containing 100 μl of the growth medium and then treated with a designated series of doses of JQ1 or SSB1 for 48 h. The cytotoxic effect of drugs was determined by 3-(4,5-dimethylthiazol-2-yl)-2,5-diphenyltetrazolium bromide (MTT) assay. To determine the combination index (CI), each drug was tested individually and at fixed ratio combinations across 2-fold serial dilutions. The IC_50_ of individual drugs and the CI value of their combination was calculated using the software COMPUSYN (CI values <1 indicate synergistic effects, =1 indicate additive effects, and >1 indicate antagonistic effects).

### Colony formation assay

Cells were incubated with JQ1, SSB1, or their combination at half of the IC_50_ value for 24 h and then plated into 6-well plates at a density of 250 cells/well for 7-d culturing, subsequently. Colonies were fixed with a 4% paraformaldehyde fixative and evaluated under a microscope following the staining of viable cells with 0.5% crystal violet solution. Then, the crystal violet stain was dissolved using 1.5 ml of dilute acetic acid solution (10%, *v*/*v*), and the optical density was measured at 590 nm.

### Cell cycle and apoptosis analysis

The cell cycle analysis was performed using Cell Cycle and Apoptosis Analysis Kit (Beyotime). Briefly, cells were incubated with JQ1, SSB1, or their combination for 48 h at a concentration of half of IC_50_. Then, cells were harvested and fixed in 70% ethanol for 3 h, and about 1 × 10^6^ cells from each sample were stained with propidium iodide (PI)/RNase staining solution in the dark for half an hour. Flow cytometry analysis was performed using an LSRFortessa flow cytometer (BD Biosciences) to assess the cell cycle distribution. For the apoptosis evaluation, cells were plated into 6-well plates and incubated with JQ1, SSB1, and their combination for 48 h. The cells were harvested, washed, and stained by allophycocyanin–annexin V and PI in the dark for 15 min. All stained samples were immediately analyzed by the flow cytometer (LSRFortessa, BD Biosciences).

### Real-time polymerase chain reaction analysis

To measure changes in the messenger RNA (mRNA) level, cells were treated with JQ1, SSB1, and their combination at a concentration of half of IC_50_. Treated cells were rinsed and lysed, followed by isolation of total RNA using RNeasy Mini Kit. The total RNA was converted to complementary DNA by a multiscribe reverse transcription kit (Applied Biosystems), and real-time quantitative reverse transcription polymerase chain reaction (qRT-PCR) was performed using SYBR Green qPCR Master Mix on a LightCycler 480 instrument (Roche) using primers (Table S1).

### Cell migration and invasion study

Cell migration was assessed using scratch wound healing assays. Cells were plated and kept cultured to form a confluent monolayer in 6-well plates, and a scratch line was created by a 200-μl pipette tip, followed by gentle washing to remove detached cells. The monolayers were then treated with JQ1, SSB1, or their combinations at a concentration of half of IC_50_. Images of the scratch area were captured at 24 h post-scratching using a brightfield inverted microscope (Zeiss, Germany).

Cell migration was also investigated by the Boyden chamber assay. Cells were incubated with JQ1, SSB1, and their combination for 24 h with a concentration of IC_50_ and then collected, counted, and seeded into the upper chamber of a 24-well transwell insert (3.0-μm pore size) at the same cell number (1 × 10^5^ cells/well). After 24-h incubation with FBS-free medium, nonmigrated cells on the upper surface were gently wiped off with a cotton swab. Migrated cells on the lower membrane were fixed in 70% ethanol for 30 min, stained with 0.1% crystal violet, and imaged under a brightfield inverted microscope. Migrated cells were counted to calculate the inhibition percentage.

Cell invasion was determined using the Boyden chamber assay. Transwell inserts were coated with 50 μl of Matrigel and further incubated at 37 °C for 30 min to form a thin gel layer. Cells were treated with JQ1, SSB1, and their combination for 24 h with a concentration of IC_50_ and then collected and seeded into the upper chamber of a 24-well transwell insert (3.0-μm pore size) at the same cell number (1 × 10^5^ cells/well). After 24-h incubation, the invading cells were fixed and stained in the same way as described in the migration assay and imaged using a brightfield inverted microscope.

### CD15^+^ and CD133^+^ MB stem cell analysis

CD15^+^ DAOY and CD15^+^ ONS-76 cells were plated into 6-well plates and allowed to attach overnight. Then, cells were incubated with JQ1, SSB1, and their combination for 48 h. After incubation, cells were harvested, washed, and stained with BV650 Mouse Anti-Human CD15 and PE Anti-human CD133 Antibody, followed by analysis by flow cytometry (LSRFortessa, BD Biosciences).

### In vitro BBB model and cellular uptake investigation

The in vitro BBB penetration ability of NPs was evaluated using the transwell assay. HBMECs were plated into the transwell upper chamber for monolayer formation. The transepithelial electrical resistance was determined until a value of 200 Ω·cm^2^ was achieved. MB cells were seeded into 6-well plates at a density of 1 × 10^5^ cells per well and incubated overnight to allow attachment. Then, the prepared HBMEC monolayer in the transwell insert was placed on the plate, and FBS-free culture medium containing Cy5.5-labeled nontargeted NPs or Try-derived NPs with a concentration of 100 μg/ml was added in the upper chamber. The cells in plates and transwell inserts were cultured for 6 h, collected, rinsed, and analyzed by flow cytometry and confocal laser scanning microscopy (fixed and stained with 4′,6-diamidino-2-phenylindole [DAPI] and fluorescein isothiocyanate–OCT2 antibody).

### Orthotopic MB mouse model

For orthotopic MB model establishment, luciferase-expressing DAOY cells were implanted into the cerebellum area of NSG mice as described below. NSG mice were anesthetized and positioned on a stereotaxic frame, followed by exposure of the skull. A small burr hole was drilled in the skull 1 mm posterior to the lambdoid suture and 1 mm lateral to the sagittal suture over the cerebellum. A total of 10 μl of cell suspension in phosphate-buffered saline (PBS; containing 1 × 10^5^ cells) was implanted into the cerebellum at a rate of 1 μl/min. The tumor growth was continuously monitored for 3 weeks using an IVIS imaging instrument.

### In vivo BBB permeability of Try-derived NPs

The BBB penetration ability of Try-derived NPs was assessed in both normal NSG mice and orthotopic MB NSG mice. These mice were euthanized 2 h post-intravenous injection of Cy5.5-labeled Try-derived NPs and nontargeted NPs. The brain tissues were collected, embedded using OCT, and sliced into 10-μm-thick sections on glass slides. Coverslips were mounted onto the glass slides using ProLong Gold antifade reagent containing DAPI for nuclear staining. Fluorescence images were captured with an LSM710 confocal laser scanning microscope equipped with a ×40 oil immersion objective and corresponding filters.

### Biodistribution of NPs

The biodistribution of JQ1- and SSB1-loaded Try-derived NPs and nontargeted NPs was determined in MB NSG mice. Drug-loaded NPs were injected intravenously; blood and major organs were collected at 6 and 24 h postinjection. The drug concentration in major organs was measured using liquid chromatography–tandem mass spectrometry (LC–MS/MS) (4000 QTRAP, AB, Sciex Inc.) with the following steps: Organ tissues were weighed, homogenized in 1 ml of water, and spiked with OTX-015 as an internal standard. Subsequently, 1 ml of acetonitrile was added for protein precipitation. The mixture was centrifuged, and the supernatant was evaporated to dryness at 37 °C under airflow. The residue was reconstituted with mobile phase and analyzed using LC–MS/MS assay. Shimadzu LC-20 AT furnished with an RP18 column (Xterra, Waters) was used for separation with the following conditions: mobile phase of acetonitrile:water (60:40, *v*/*v*), flow rate of 1 ml/min, and column temperature of 30 °C. LC–MS/MS data acquisition was performed by using the Analyst software, and the mass spectrometer was operated in positive mode with selected multiple reaction monitoring for JQ1 (*m*/*z* 457.0 → 401.0), OTX-015 (*m*/*z* 492.0 → 353.0), and SSB1 (*m*/*z* 780.0 → 617.0).

### Anti-tumor ability

Orthotopic MB NSG mice were randomly assigned to 7 groups (*n* = 5) and were administered the following treatments: PBS, JQ1-loaded nontargeted NPs, SSB1-loaded nontargeted NPs, JQ1 + SSB1-loaded nontargeted NPs, JQ1-loaded Try-derived NPs, SSB1-loaded Try-derived NPs, and JQ1 + SSB1-loaded Try-derived NPs. Each treatment was delivered via intravenous injection every 3 d at a dose of 20 mg/kg per compound. Body weight and MB tumor growth were monitored during the treatment. After the treatment period (total of 7 times of administration), mice were euthanized to dissect the brain and other major organs. Brain samples were fixed, sliced, and stained with hematoxylin and eosin (H&E) and subjected to immunohistochemical staining for key proteins.

### Statistical analysis

Statistical analysis was conducted by GraphPad Prism 8.0 (IBM Corp., Armonk, NY, USA). Data are displayed as mean ± standard deviation with replicates at least 3 times. Differences between 2 groups were analyzed using the *t* test, and those of more than 2 groups were analyzed using one-way analysis of variance using Tukey’s test. The data were considered significant when **P < 0.01 and *P < 0.05; ns means no significance.

## Results

### SSB1 inhibits MB cell proliferation and GLI expression more efficiently than GDC-0449

The viability of DAOY (SHH MB subtype) and D341 MED (group 3 MB subtype) cells after incubation with different concentrations of SSB1 was calculated using the MTT assay. SSB1 decreased the cell viability at a lower dose on both MB cell lines than that of GDC-0449 at various drug concentrations (Fig. S2A and B). The IC_50_ of SSB1 was found to be significantly lower compared to those of GDC-0449 on DAOY cell lines (4.1 μM vs. 94.5 μM) and D341 MED cell lines (5.3 μM vs. 120.4 μM), indicating that SSB1 has a higher binding affinity to the SMO receptor compared to GDC-0449.

To determine whether SSB1 shows its effect by inhibiting SMO, we knocked down *SMO* expression by transfection of siSMO using Lipofectamine 2000 before treating cells with SSB1. *SMO* silencing in DAOY cells with siSMO was dose dependent, with almost 80% *SMO* silencing with 25 nM siSMO as determined by real-time PCR (Fig. S3). SMO knockdown in DAOY cells exhibited resistance to SSB1 treatment with a significant enhancement in IC_50_ of 19.9 μM. In contrast, scrambled small-interfering-RNA-transfected cells showed no effect on the cell-killing potential of SSB1 (Fig. S2C). Moreover, JQ1-treated *SMO* knockdown MB cells have a similar IC_50_ value as SMO-expressing MB cells (Fig. S4). These results indicate that JQ1 could overcome SMO mutation in MB treatment.

### The combination of SMO and BRD4 inhibitors has a synergistic effect in MB killing and inhibiting colony formation

To determine whether the combination of SMO and BRD4 inhibitors could improve MB treatment, we first determined the cell viability and colony formation ability of MB cells in the presence of SSB1 and JQ1. After 48-h incubation, JQ1 and SSB1 significantly decreased DAOY and D341 Med cell viability in a dose-dependent manner (Fig. S2D and E). The IC_50_ of JQ1 were 4.2 μM in DAOY cells and 402 nM in D341 Med cells, whereas SSB1 has IC_50_ values of 4.1 and 5.3 μM for DAOY and D341 Med cells, respectively. We further analyzed the synergistic effects of these 2 drugs by incubating DAOY and D341 Med cells with different drug ratios. JQ1 and SSB1 combination significantly decreased cell viability in of both these cell lines and showed a synergistic effect on cell killing, with the CI value lower than 1 at the ratios marked as a red cycle in these figures (Fig. S2F and G).

A colony formation assay was also carried out to determine whether SSB1 and JQ1 synergistically inhibited colony formation when DAOY and D341 MED cells were incubated with their combination. There was significant inhibition of colony formation after JQ1 or SSB1 incubation, and the inhibitory effect was the highest for the combination therapy in both these cell lines (Figs. S2H and I and S5).

### JQ1 and SSB1 synergistically inhibit GLI expression, arrest the cell cycle, and promote the apoptosis of MB cells

**Fig. 1.**
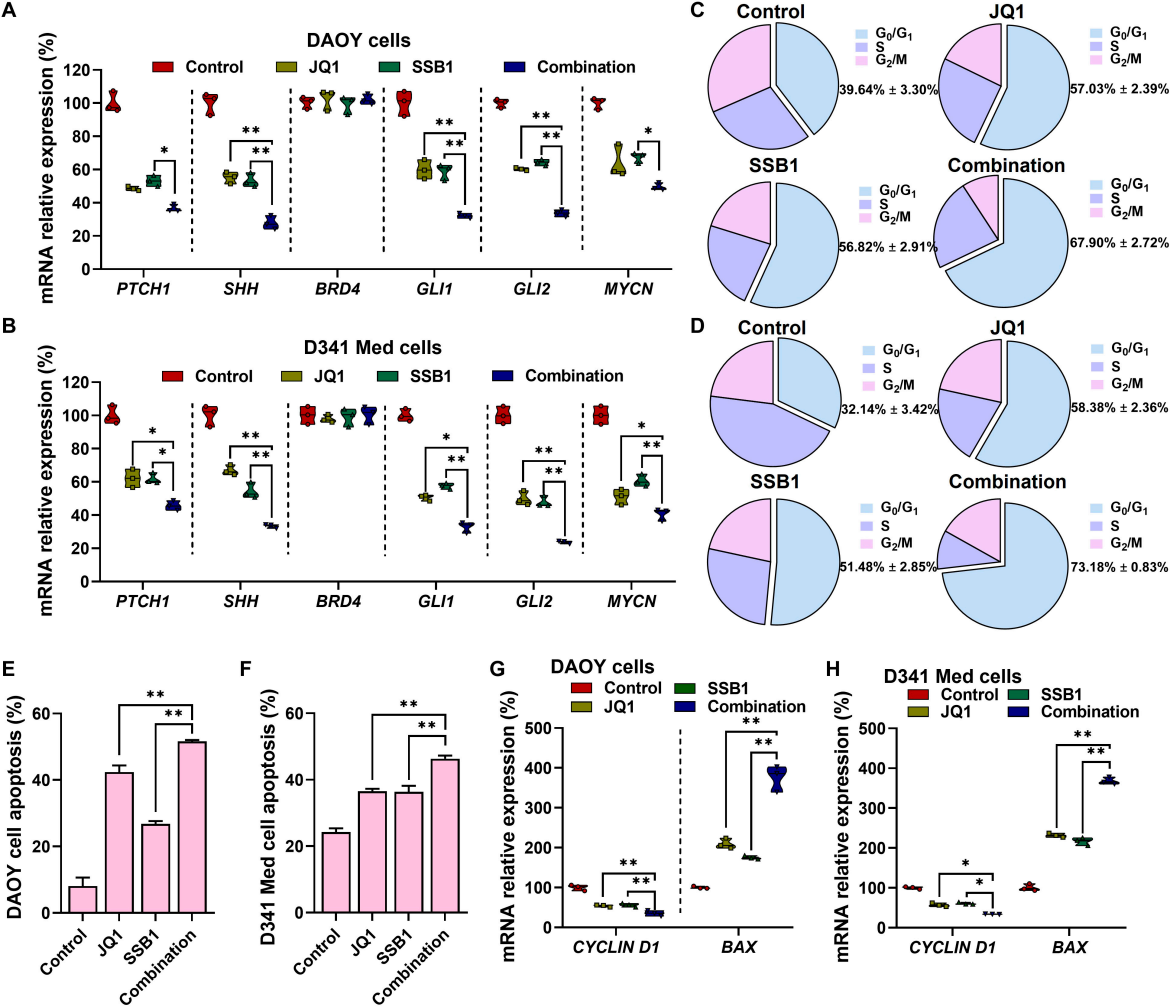
JQ1 and saikosaponin B1 (SSB1) synergistically inhibit GLI expression, arrest cells in the G_0_/G_1_ phase, and promote the apoptosis of medulloblastoma (MB) cells. (A and B) The inhibition of *GLI1/2*, *PTCH1*, *SHH*, and *MYCN* messenger RNA (mRNA) expression in DAOY and D341 Med cells with JQ1 and SSB1 treatment for 48 h was determined by real-time quantitative reverse transcription polymerase chain reaction (qRT-PCR). (C and D) Cell cycle of DAOY and D341 Med cells treated with JQ1, SSB1, and their combination for 48 h. (E and F) Cell apoptosis of DAOY and D341 Med cells treated with JQ1, SSB1, and their combination for 48 h. (G and H) *CYCLIN D1* and *BAX* mRNA expression in DAOY and D341 Med cells after incubation with JQ1 and SSB1 combination (*P < 0.05; **P < 0.01).

GLI proteins serve as crucial transcription factors within the Hh signaling pathway. Here, we confirmed SHH pathway inhibition by JQ1 and SSB1 by measuring the Hh pathway components at mRNA levels. As shown in Fig. 1A and B, the combination of JQ1 and SSB1 significantly down-regulated key genes such as *GLI1*, *GLI2*, *SHH*, *PTCH1*, and *MYCN* compared to JQ1 or SSB1 treatment alone in both DAOY and D341 Med cells, indicating that JQ1 and SSB1 combination could synergistically inhibit *GLI* expression.

GLI is known to regulate the cell cycle by elevating cyclin D, a key factor essential for G_1_ phase progression. After treatment with a JQ1 and SSB1 combination, the percentage of cells in the G_1_ phase was significantly increased (70.9% ± 0.7% in DAOY and 73.7% ± 3.0% in D341 MED) compared to that in the control (40.6% ± 1.3% in DAOY and 35.1% ± 2.0% in D341 MED), and for monotherapy, it was 61.0% ± 2.4% for JQ1 and 54.8% ± 2.9% for SSB1 in DAOY cells, while it was 59.7% ± 1.9% for JQ1 and 51.3% ± 2.2% for SSB1 in D341 MED cells (Fig. 1C and D and Fig. S6). Furthermore, apoptotic cells were quantified and analyzed by annexin V/PI double staining followed by flow cytometry (Fig. 1E and F and Fig. S7). JQ1 and SSB1 could effectively induce apoptosis in DAOY cells (49.3% ± 1.5% and 47.0% ± 0.7%, respectively) compared to the control (2.3% ± 0.3%). In D341 MED cells, the apoptotic cells after treatment with JQ1 or SSB1 increased to 29.7% ± 0.3% and 27.4% ± 0.2%, respectively, compared to those in the control cells (15.9% ± 0.4%). The combination of these drugs further increased the apoptotic proportion (66.5% ± 0.8% for DAOY cells and 46.3% ± 1.0% for D341 MED cells), indicating that combination therapy could more effectively inhibit the tumor cell growth.

Next, cell-cycle- and apoptosis-related genes were investigated by measuring the associated molecules using the qRT-PCR assay. As shown in Fig. 1G and H, there is an up-regulation in the mRNA levels of *CYCLIN D* and the pro-apoptotic gene *BAX*, which confirms that both JQ1 and SSB1 could inhibit MB cell proliferation by modulating the key regulators in the cell cycle and cell apoptosis.

### The combination of SSB1 and JQ1 efficiently reduces MB stem cell proliferation

**Fig. 2.**
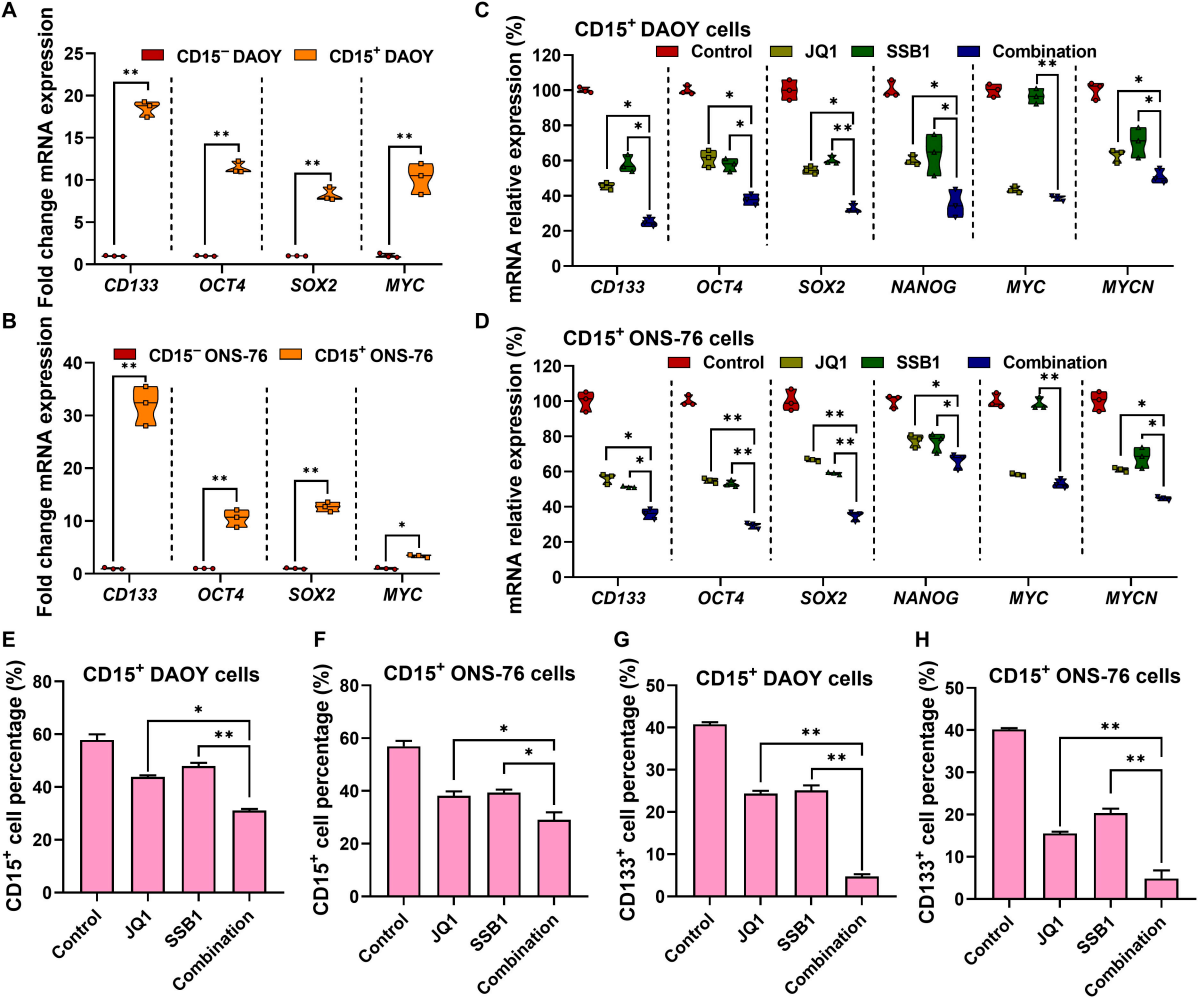
JQ1 and SSB1 synergistically down-regulate MB stem cell markers and reduce frequency. (A and B) mRNA levels of stem cell markers in CD15^+^ MB and CD15^−^ MB cells. (C and D) The mRNA levels of stem cell markers in CD15^+^ DAOY and CD15^+^ ONS-76 cells after incubation with JQ1 and SSB1 for 48 h were determined by real-time RT-PCR. (E and F) CD15^+^ MB stem cell frequency reduced after incubation with JQ1, SSB1, and their combination. (G and H) CD133 expression on CD15^+^ MB stem cells after incubation with JQ1, SSB1, and their combination (*P < 0.05; **P < 0.01).

It has been reported that SHH MB is driven by proliferative CD15^+^ CSCs, which induce cell proliferation, therapy resistance, and metastasis. Here, we isolated CD15^+^ CSCs from SHH MB cell lines DAOY and ONS-76 using a flow cytometer after incubating them with BV650-CD15 antibody. Both CD15^+^ cells and CD15^−^ cells were harvested, and CSC markers were determined using the qPCR assay. As shown in Fig. 2A and B, the mRNA levels of stem cell markers such as *CD133*, *OCT4*, *SOX2*, and *MYC* were significantly higher in CD15^+^ ONS-76 and DAOY cells compared to those in CD15^−^ cells, indicating that CD15^+^ ONS-76 and DAOY cells show a stem-like phenotype.

The stemness of CSCs is related to specific signaling pathway activation, including the SHH pathway. The activation of GLI could induce the expression of key genes such as *OCT4*, *SOX2*, and *NANOG* to maintain the stemness of CSCs. As JQ1 and SSB1 synergistically reduce *GLI* expression, we evaluated the treatment effects of these 2 drugs to inhibit CSCs’ proliferation and differentiation. After being treated with JQ1 and SSB1 for 48 h, the mRNA levels of stem cell markers such as *CD133*, *OCT4*, *SOX2*, *NANOG*, and *MYC* were significantly decreased compared to those of control and monotherapy (Fig. 2C and D). Moreover, the CD15 and CD133 expression on the cell membrane surface was decreased after JQ1 and SSB1 co-incubation and was analyzed using a flow cytometry assay, which implies that the percentage of MB cells with a stem phenotype was reduced due to the treatment (Fig. 2E to H). The key genes for CSCs to maintain stemness, such as *OCT4*, *SOX2*, and *NANOG*, were also significantly decreased after the combination treatment (Fig. 2C and D), further implying that the stemness of CSCs was reduced and different from that of normal tumor cells.

**Fig. 3.**
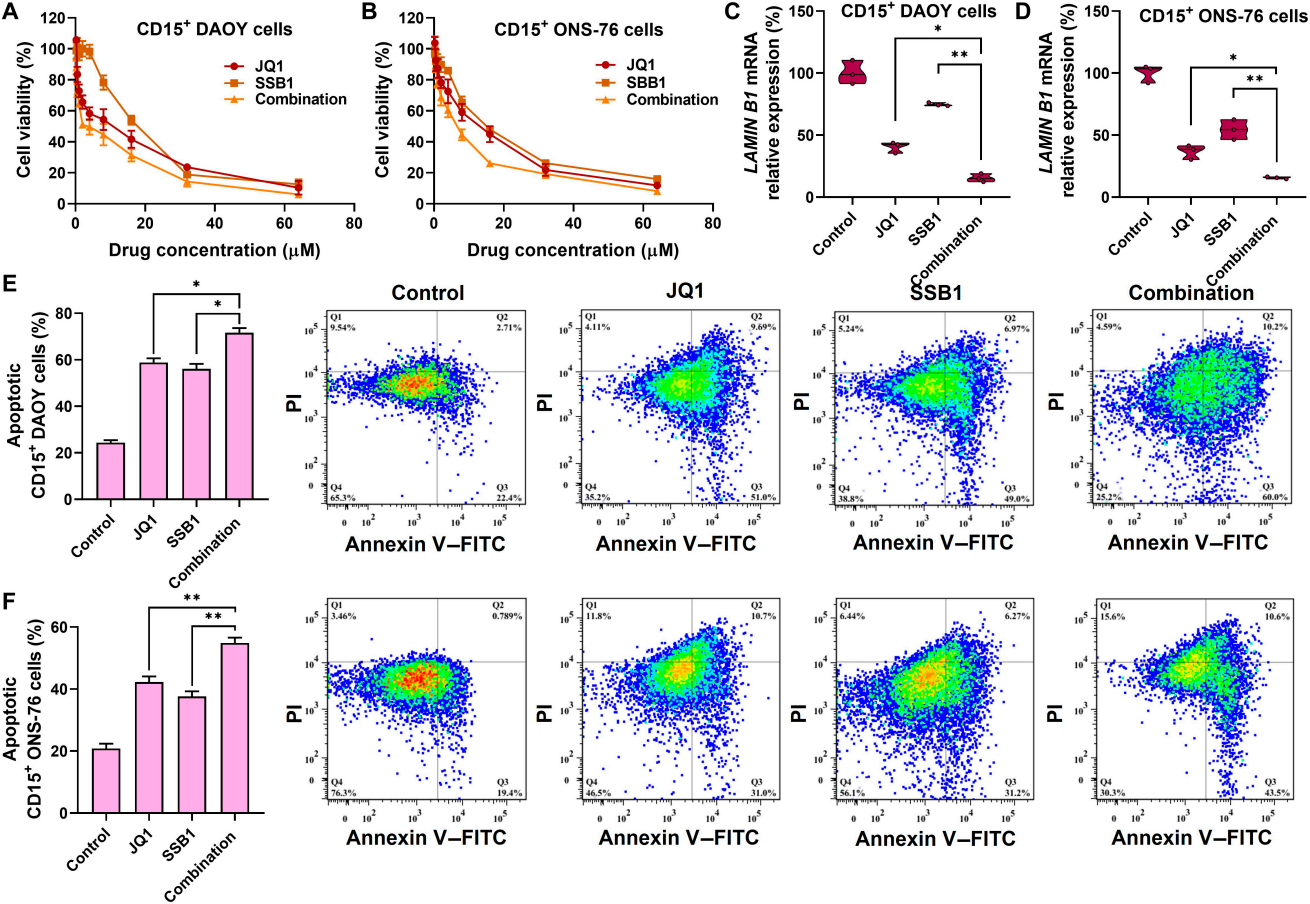
JQ1 and SSB1 synergistically increase MB stem cell apoptosis. (A and B) JQ1 and SSB1 inhibit CD15^+^ MB stem cell viability in a dose-dependent manner. (C and D) mRNA expression of senescent markers in CD15^+^ MB stem cells with JQ1 and SSB1 incubation for 48 h. (E and F) The apoptosis of CD15^+^ MB stem cells after incubation with JQ1, SSB1, and their combination, as well as the representative flow cytometry scatter plots. PI, propidium iodide; FITC, fluorescein isothiocyanate.

We further investigated the cytotoxicity of JQ1 and SSB1 on MB stem cells. As shown in Fig. 3A and B, both JQ1 and SSB1 show increased IC_50_ values on CD15^+^ DAOY (4.67 μM for JQ1 and 14.97 μM for SSB1) and CD15^+^ ONS-76 stem cells (11.41 μM for JQ1 and 10.80 μM for SSB1) compared to normal DAOY cells (4.2 μM for JQ1 and 4.1 μM for SSB1) and ONS-76 cells (5.1 μM for JQ1 and 4.8 μM for SSB1, data not shown), indicating the drug resistance of stem cells. The combination of JQ1 and SSB1 could effectively and synergistically reduce the viability of MB stem cells compared to monotherapy. We further measured *LAMIN B1*, a senescent biomarker, and *CLEAVED CASPASE 3* expression in MB stem cells after treatment. The *LAMIN B1* mRNA expression in CD15^+^ MB stem cells was significantly decreased after JQ1 treatment (Fig. 3C and D), indicating a BET inhibitor (BETi) with the potential to lead to stem cell senescence. Next, the combination of JQ1 and SSB1 could also inhibit CD15^+^ MB stem cell proliferation not only due to cell senescence but also by inducing cell apoptosis. As shown in Fig. 3E and F, the combination of JQ1 and SSB1 synergistically increases the apoptosis of MB stem cells compared to JQ1 and SSB1 monotherapy, analyzed using flow cytometry (CI value <0.8).

### SSB1 and JQ1 synergistically inhibit MB metastasis

**Fig. 4.**
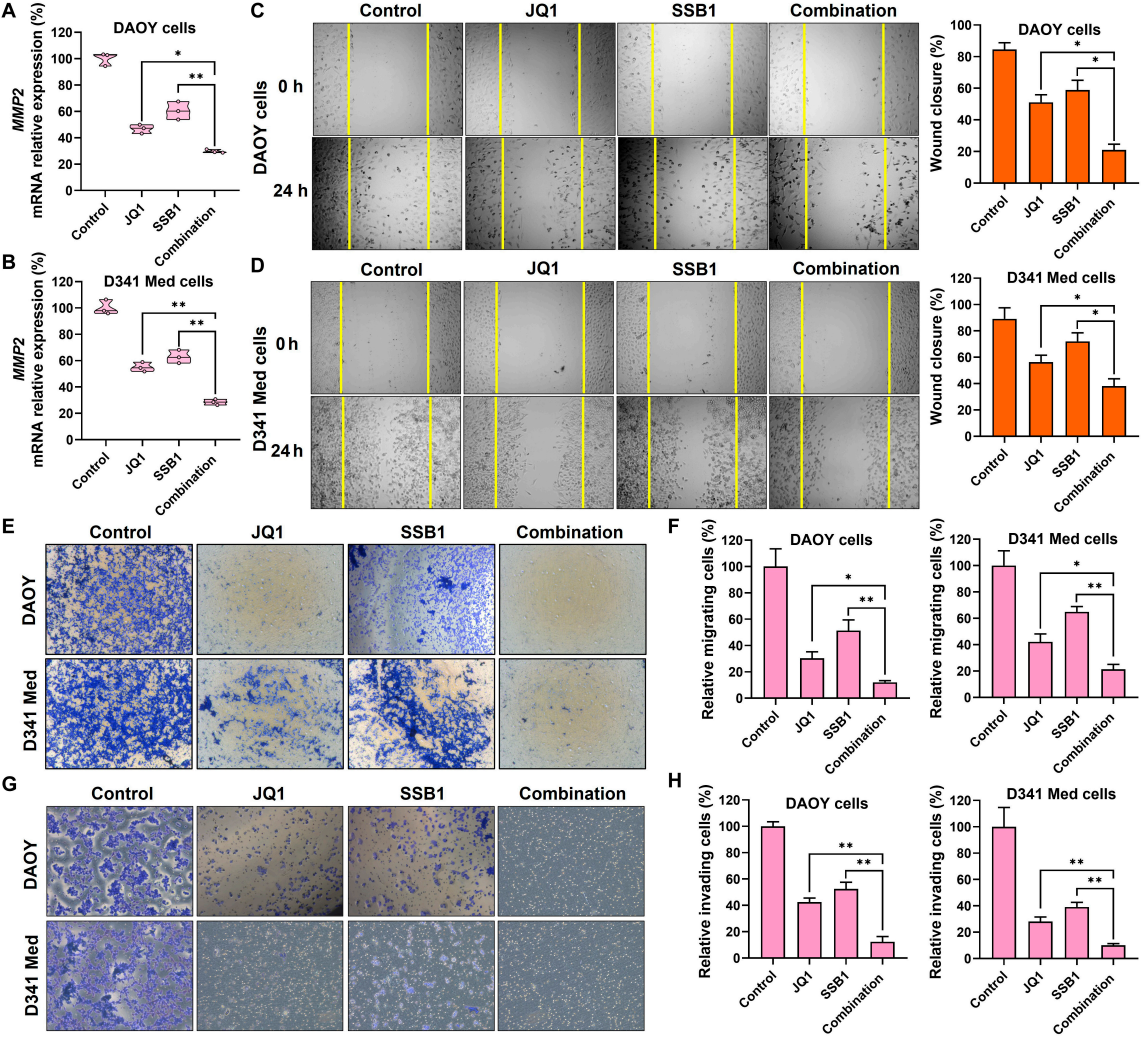
JQ1 and SSB1 synergistically down-regulate MMP2 and inhibit MB metastasis. (A and B) JQ1 and SSB1 down-regulate *MMP2* mRNA expression in MB cells. (C and D) The microscope images and quantitative analysis of DAOY and D341 Med cells after incubation with JQ1, SSB1, and their combination. The microscope images and quantitative analysis of MB cell migration (E and F) and invasion (G and H) after incubation with JQ1, SSB1, and their combination (*P < 0.05; **P < 0.01). GSH, glutathione; DAPI, 4′,6-diamidino-2-phenylindole; OCT2, organic cation transporter 2.

The metastasis of cancer is a complex multistep process, including migrating and invading tumor cells, which involves multiple genetic alterations. MMP-mediated degradation of the ECM represents a critical step in the metastatic process. MMPs produced by tumor cells could destroy the matrix barriers surrounding the tumor, helping tumor cells migrate and invade the surrounding connective tissues, blood vessels, or cerebrospinal fluid for the MB to metastasize to distant organs. Previous research has shown that MMPs exhibit elevated expression across a broad spectrum of tumors, including MB. MMP2 is one of the key promoters related to the migration and invasion of MB. We first measured *MMP2* levels in normal brain cells and MB cells. The mRNA level of *MMP2* was elevated in MB cell lines in SHH MB and group 3 MB compared to that in normal brain cell lines (Fig. S8), consistent with previous research. MMP2 was directly regulated by BRD4 and PI3K/AKT signaling. JQ1 could directly reduce BRD4 transcription, and SSB1 could inhibit *GLI2* expression, another critical transcription factor of MMP2. Therefore, we speculated that JQ1 and SSB1 could synergistically reduce MMP2 expression and further reduce the migration and invasion of MB cells. As shown in Fig. 4A and B, the mRNA level of MMP2 was significantly reduced after JQ1 and SSB1 combination treatment.

Next, we investigated the migration and invasion ability of MB cells after JQ1 and SSB1 combination treatment. The wound healing assay was a primary method for cell migration investigation. After creating a scratch on the MB cell monolayer, cells were incubated with JQ1, SSB1, and their combination for 24 h. The scratch wound was almost healed in the control group, with full cells migrating into the wound area, while the wound space remained after incubation with JQ1 and SSB1 alone (Fig. 4C and D). The combination exhibits limited cell migration into the wound area, which most effectively reduces cell migration in both SHH MB and group 3 MB. Further, the transwell cell migration assay was also used to confirm the migration ability of MB cells. As shown in Fig. 4E and F, the combination treatment most effectively reduced MB cell migration in both subtypes of MB compared to control and monotherapy. The cell invasion across Matrigel was further investigated. Data suggested that the combination of JQ1 and SSB1 could also inhibit both DAOY and D341 Med cell invasion, indicating the possibility of the combination inhibiting MB metastasis (Fig. 4G and H).

### Formulation and characterization of drug-loaded Try-derived NPs

**Fig. 5.**
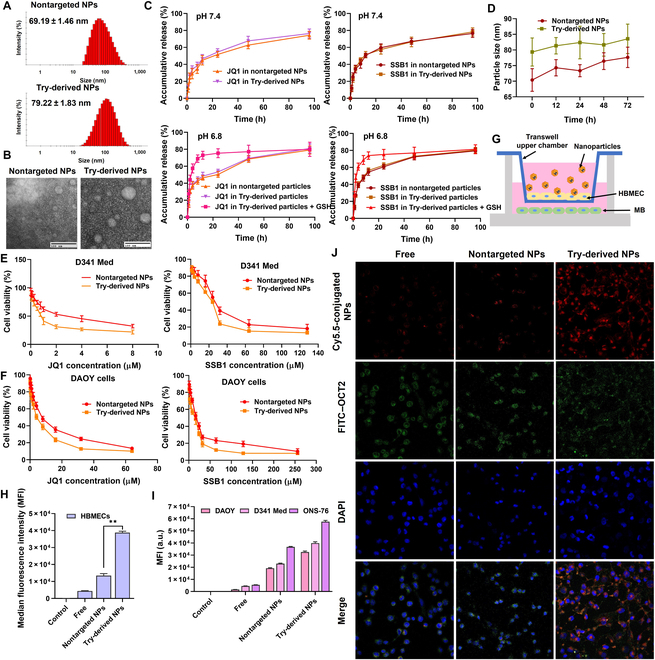
Characterization of drug-loaded nanoparticles (NPs). (A) Dynamic light scattering (DLS) of JQ1- and SSB1-loaded nontargeted NPs and tryptamine (Try)-derived NPs. (B) NP morphology analyzed by transmission electron microscopy (TEM). (C) The cumulative release profiles of JQ1 and SSB1 in nontargeted NPs and Try-derived NPs at pH 7.4 and 6.8. (D) Stability of JQ1- and SSB1-loaded nano-targeted NPs and Try-derived NPs at room temperature for 72 h. (E and F) Cell viability of MB cells after 48-h incubation with JQ1- and SSB1-loaded nontargeted NPs and Try-derived NPs. (G) Scheme of the in vitro transwell blood–brain barrier (BBB) model. (H and I) The cellular uptake of Cy5.5-decorated nontargeted NPs and Try-derived NPs in human brain microvascular endothelial cells (HBMECs) and MB cells after 4-h incubation was analyzed using flow cytometry. (J) The cellular uptake of Cy5.5-decorated nontargeted NPs and Try-derived NPs in DAOY cells was observed by confocal microscopy.

Try-Lips were synthesized following the route in Fig. S8. The structure of Try-Lips was confirmed by the ^1^H nuclear magnetic resonance spectrum (Fig. S9). JQ1- and SSB1-loaded Try-derived NPs were formulated by film hydration assay. The particle sizes of drug-loaded nontargeted NPs and Try-derived NPs were 69.19 ± 1.46 and 79.22 ± 1.83 nm (Fig. 5A), respectively, which are suitable for both BBB penetration and avoids kidney excretion to increase the long circulation in vivo. The transmission electron microscopy images confirmed the spherical shape of nontargeted NPs and Try-derived NPs with a consistent particle size (Fig. 5B). This particle size range is suitable for both BBB penetration and avoids kidney excretion to increase the long circulation in vivo. The DL capacity and encapsulation efficiency of JQ1 were 9.3% ± 0.3% and 90.3% ± 2.8%, respectively, whereas those of SSB1 were 5.1% ± 0.1% and 87.7% ± 1.1%, respectively, indicative of the acceptable DL of JQ1 and SSB1 in Try-derived NPs.

Release curves are indicative of the little effects of Try decoration on drug release compared to those of nontargeted NPs in 48 h at pH 7.4 (Fig. 5C). The cumulative release was not significantly improved in the weak acid environment at pH 6.8 either, whereas the drug was rapidly released to the medium from Try-derived NPs in the presence of glutathione (GSH) at pH 6.8 compared to that under conditions without GSH, suggesting that the drug-loaded Try-derived NPs exhibited a reductive responsive drug release, which could target released anti-tumor agents only in tumor cells with extremely high intracellular GSH levels (Fig. 5C). In the stability investigation, the particle sizes of drug-loaded formulations remained stable within 72 h of storage at 4 °C (Fig. 5D), suggesting the acceptable stability of Try-derived NPs. The cytotoxicity of drug-loaded Try-derived NPs was evaluated in both SHH MB and group 3 MB cells. Try-derived NPs significantly reduced cell viability compared to nontargeted NPs in all MB cell lines at the same dosage, indicating the higher uptake of drug-loaded Try-derived NPs by MB cells compared to nontargeted NPs (Fig. 5E and F and Fig. S10).

### Try-derived NPs show BBB permeability and increase drug accumulation in the brain

To assess BBB permeability, we established an in vitro BBB model using the transwell assay (Fig. 5G and Fig. S11). The cellular uptake of Cy5.5-decorated DSPE-PEG_2k_ was incorporated into the preparation of Try-derived NPs and nontargeted NPs for the cellular uptake investigation. After adding them to the upper chamber, they were incubated with HBMECs for 6 h. Cy5.5-labeled Try-derived NPs exhibited the highest fluorescence intensity in HBMECs, suggesting excellent cellular uptake by BBB endothelial cells (Fig. 5H). Similar data were observed in 3 types of MB cell lines that adhered in the lower chamber, indicating that the Cy5.5-decorated Try-derived NPs improved BBB crossing compared to nontargeted NPs (Fig. 5I). We further used confocal microscopy to confirm the cellular uptake of NPs in DAOY cells. As shown in Fig. 5J, the intracellular Cy5.5 signals were significantly brighter than those of the free group and nontargeted NPs, confirming the stronger cellular uptake of Try-derived NPs in MB cells. Moreover, we further located the OCT2 expression on MB cells and observed the signal overlaps of OCT2 and Try-derived NPs, which led us to speculate that the Try-derived NPs were taken up into MB cells through OCT2-mediated assay.

**Fig. 6.**
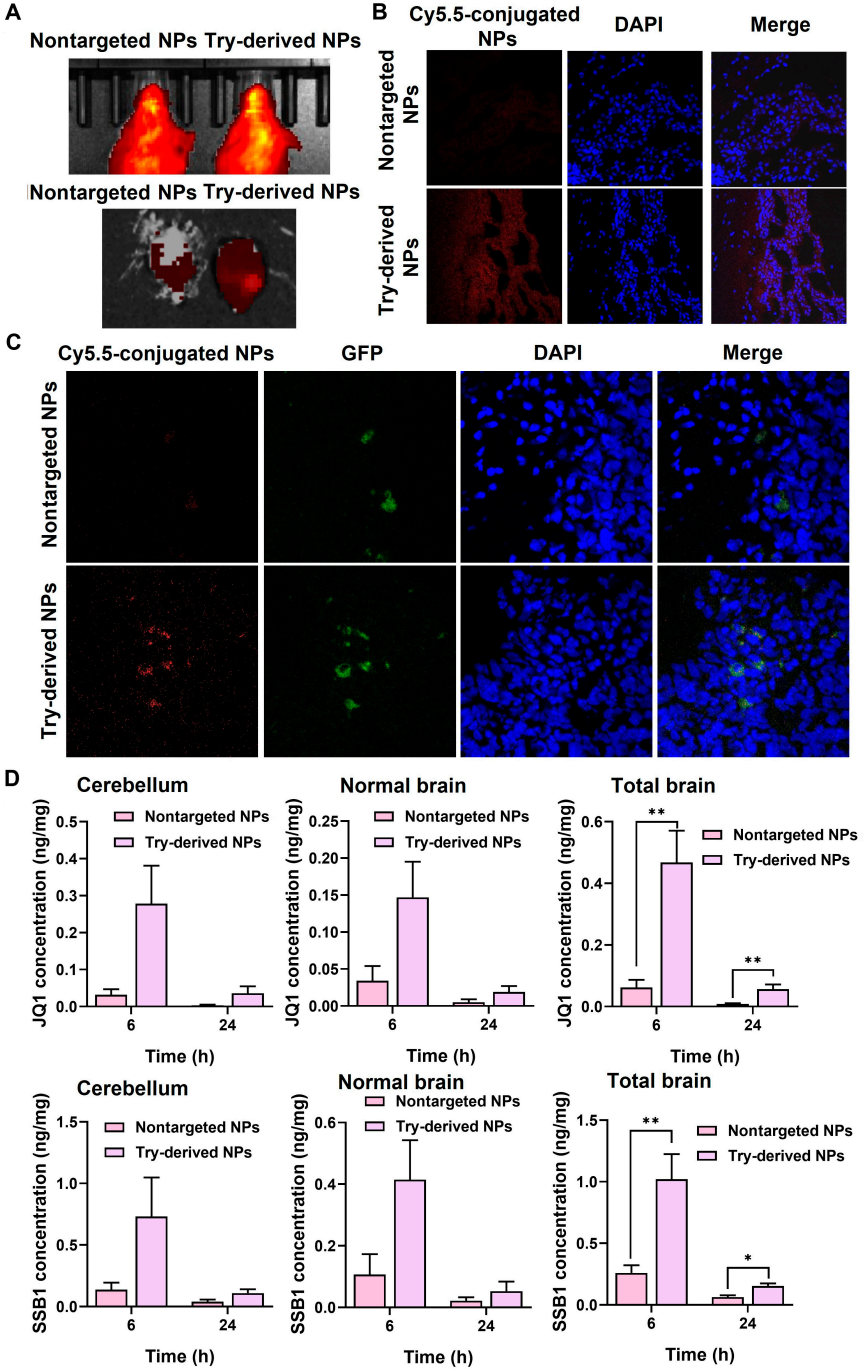
Try-derived NPs significantly enhance BBB permeability and drug biodistribution after systemic administration. (A and B) Try-derived NPs conjugated with Cy5.5 significantly increase brain permeability compared to nontargeted NPs in healthy NSG mice analyzed using the IVIS system and confocal microscopy. (C) Try-decorated NPs conjugated with Cy5.5 significantly increase MB tumor cellular uptake compared to nontargeted NPs in MB-bearing NSG mice. (D) JQ1 and SSB1 concentrations in the brains of orthotopic MB tumor-bearing NSG mice at 6 and 24 h post-systemic administration of drug-loaded Try-derived NPs or nontargeted NPs at a dose of 10 mg/kg (*P < 0.05; **P < 0.01). GFP, green fluorescent protein.

We further investigated the BBB crossing ability of Try-derived NPs in vivo in both healthy NSG mice and orthotopic MB-bearing mice. In healthy mice, the Try-derived NP group exhibited higher Cy5.5 signals in the brain than the nontargeted NP group 2 h post-administration, which was observed using IVIS (Fig. 6A). The accumulation of NPs in the brain was further confirmed using a confocal microscope by analyzing the mouse brain sections. Stronger Cy5.5 signals were observed in the brain section of healthy mice injected with Try-derived NPs than in those injected with nontargeted NPs, also indicative of Try decoration improving the BBB permeability of NPs (Fig. 6B).

Moreover, we evaluated the MB-targeting ability of Try-derived NPs in orthotopic MB tumor-bearing NSG mice. Mouse brains were also extracted to prepare frozen slides for observation using a confocal microscope. A similar phenomenon was observed in orthotopic MB tumor-bearing mice. Cy5.5 signals were located and overlapped with green fluorescent protein green signals, which belong to the MB cells in mouse brain sections, after injecting Try-derived NPs, but were hardly observed in nontargeted NP groups, which confirmed the ability of Try-derived NPs in targeting MB in vivo (Fig. 6C).

The biodistribution of JQ1 and SSB1 in orthotopic MB-bearing mice was measured using LC–MS. Try-derived NPs could efficiently cross the BBB and significantly increase JQ1 and SSB1 accumulation in the cerebellum 6 h (0.28 ± 0.10 ng/mg for JQ1 and 0.73 ± 0.32 ng/mg for SSB1) and 24 h (0.036 ± 0.019 ng/mg for JQ1 and 0.109 ± 0.032 ng/mg for SSB1) after injection compared to nontargeted NPs (0.032 ± 0.015 ng/mg for JQ1 and 0.137 ± 0.057 ng/mg for SSB1 at 6 h and 0.003 ± 0.002 ng/mg for JQ1 and 0.039 ± 0.018 ng/mg for SSB1 at 24 h) (Fig. 6D), confirming that Try-derived NPs prolonged drug accumulation in MB.

### JQ1 and SSB1 combination exerts superior anti-MB efficacy in the orthotopic MB mouse model via Try-derived NP delivery

**Fig. 7.**
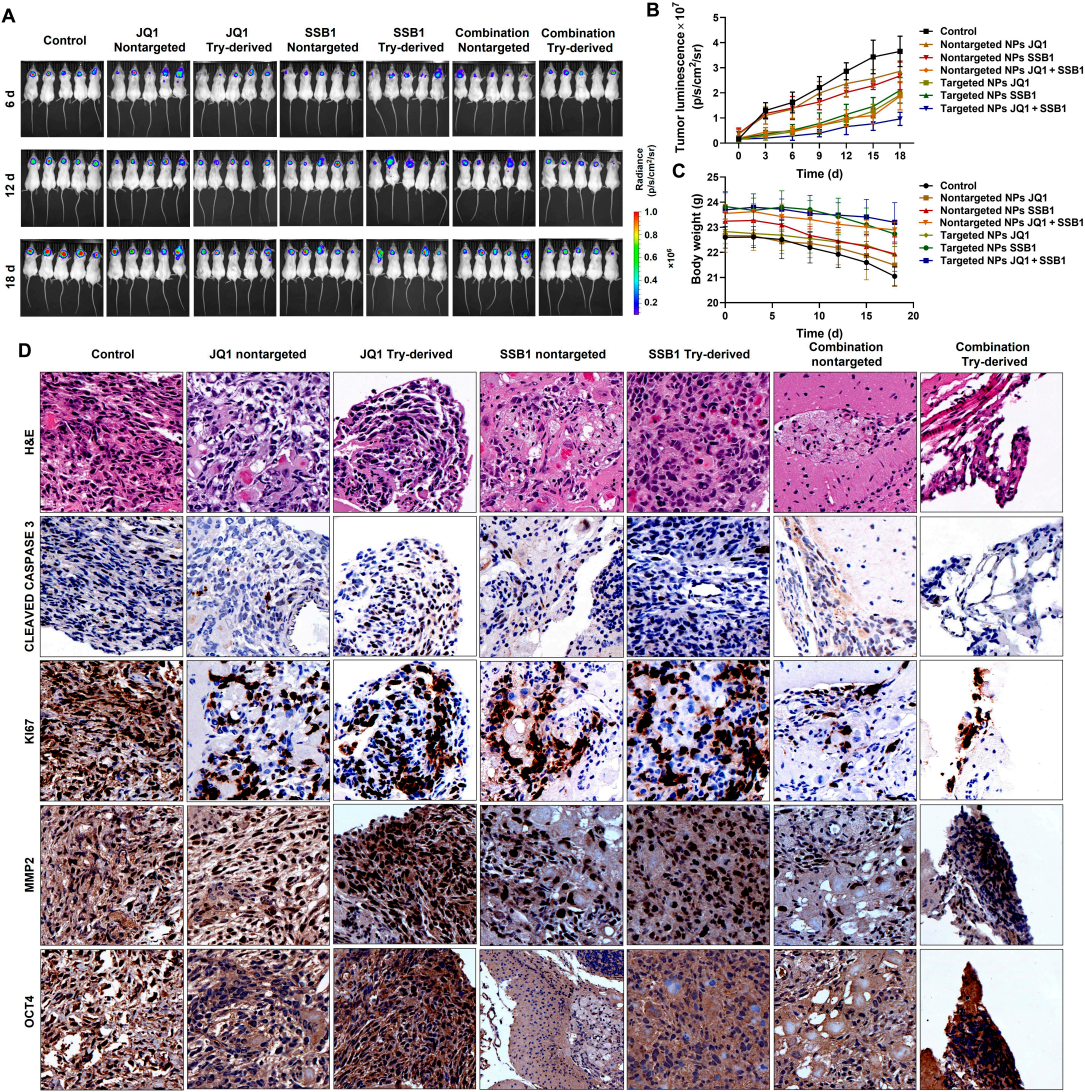
Anti-tumor efficacy of targeted NPs loaded with JQ1 and SSB1 after systemic administration into orthotopic MB-bearing NSG mice. (A) Bioluminescence images and (B) quantitative analysis of IVIS signal intensity (photons/s/cm^2^/sr) of MB tumors at different times. (C) Average mouse body weight changes during treatment. (D) Representative microscopic pictures of hematoxylin and eosin (H&E) and immunohistochemical staining of MB tissues for OCT4, MMP2, KI67, and CLEAVED CASPASE 3. Scale bar 100 μm, inset image ×20.

The therapeutic efficiency of the JQ1 and SSB1 combination on SHH MB was investigated in the tumor-bearing NSG mouse model. The bioluminescence signals of the MB tumor were significantly reduced in mice injected with a combination loaded into Try-derived NPs compared to those in mice injected with nontargeted NPs (Fig. 7A and B). Consistent with previous data, Try-derived NPs exhibited superior therapeutic efficiency due to strong BBB permeability and MB-targeting ability compared to nontargeted NPs. The body weight of mice injected with Try-derived NPs showed no significant decrease (Fig. 7C), indicating that Try-derived NPs are ideal delivery systems with low systemic toxicity. Brain tissue sections from each treatment group were subjected to H&E staining and immunohistochemical analysis (Fig. 7D). The H&E staining confirmed that the MB area was significantly reduced after JQ1 and SSB1 treatment. Reduced proliferation marker Ki67 staining and observed apoptotic marker cleaved caspase-3 also confirm that the combination could effectively inhibit MB progression. Moreover, the MMP2 staining was also reduced after treatment, indicating the potential of the combination of the BRD4 inhibitor and SMO inhibitor in preventing MB metastasis. The expression of stem-related regulator OCT4 was also reduced after treatment, suggesting that the combination could further inhibit the activation of MB stem cells.

## Discussion

MB ranks among the most prevalent malignant central nervous system tumors, generated in the pediatric cerebellum. The therapeutic strategy of MB, including surgery, radiation, and chemotherapy, could limitedly improve the survival of patients but often at the expense of lifelong side effects such as endocrine dysfunction, ataxia, and emotional lability. Furthermore, MB exhibits a high chance of spinal metastases and also the highest probability of extraneural dissemination of all central nervous system tumors. Therefore, developing a targeting therapy strategy based on disentangling the molecular basis of MB is urgently needed. SHH MB and group 3 MB were the most malignant, with poor prognosis. IGF-II activation triggers PI3K signaling, thereby collaborating with SHH/GLI to promote the progression of SHH MB progression. Group 3 MB is mostly induced by *MYC* overexpression, which will also affect GLI, a crucial transcription factor within the SHH signaling cascade. Our previous data confirmed the overexpression of GLI, MYC, and p-PI3K/p-AKT in MB patient samples. Therefore, the SHH and MYC pathways’ co-inhibition might be more effective than monotherapy.

SSB1, a potent SMO inhibitor, was extracted and purified from the roots of the traditional medicinal herb Chinese Thorowax. SSB1, as an SMO inhibitor, binds with SMO proteins, which inhibits its activation and expression of downstream GLI and SUFU. Our evaluation confirmed the better therapeutic efficacy of SSB1 in vitro compared to that of the commercial SMO inhibitor vismodegib (GDC-0449), with a lower IC_50_ value. Previous research implies that JQ1 could inhibit both MYC- and GLI-amplified tumors by binding to the BRD4 protein and reducing *MYC* and *GLI* transcription. As a transcriptional regulator, BRD4 exerts a pivotal function in embryogenesis and tumorigenesis. During the transcription process, the mediator protein complex is recruited by BRD4 and forms super-enhancer complexes to promote RNA polymerase II activity, stimulating transcription initiation and elongation. In contrast, JQ1 could directly bind with the BRD4 protein by occluding the acetyl-lysine binding pocket unique to BET family proteins. Competitively binding with JQ1, BRD4 is displaced from chromatin and inhibit super-enhancer complex formation, which reduces the *MYC* and *GLI* oncogenes’ transcription. Therefore, SSB1 combined with JQ1 was selected to improve SHH MB and group 3 MB treatment efficiency. The MTT and colony formation assays confirmed that the JQ1 and SSB1 combination could synergistically increase cell killing compared to monotherapy. The expression of key regulators such as *GLI*, *MYCN*, *SHH*, and *PTCH1* significantly decreased after incubating with the JQ1 and SSB1 combination, indicating that the combination could synergistically regulate the SHH pathway and increase apoptosis. The therapeutic resistance of SMO inhibitors to SHH MB was observed in several clinical trials. The SMO protein loses the ability to bind to the SMO inhibitor due to the mutation. Based on our research data, JQ1, as a BETi, could also regulate the SHH pathway by directly reducing the downstream genes of SMO, which could overcome the resistance of SMO inhibitors.

CSC populations in the brain, also termed brain tumor-initiating cells (BTICs), have been detected in different subtypes of MB based on cell surface markers. Read et al. [45] and Ward et al. [46] all confirmed that CD15 could be a primitive stem-like cell marker of BTIC populations in the SHH MB mouse model. Therefore, the effects of JQ1 and SSB1 on CSCs were investigated using CD15^+^ SHH MB stem cells sorted from DAOY and ONS-76 cell lines. The transcription factors GLI1/2, as the SHH signaling pathway’s prominent effectors, could also regulate key factors such as OCT4, SOX2, NANOG, and MYCN in self-renewal and keep the stemness of CSCs. OCT4 and SOX2 have been reported to maintain CSC survival by inhibiting apoptosis and promoting cell proliferation by activating the OCT4/TCL1/AKT pathway and SOX2/ORAIL/STIM1 pathway, respectively. NANOG could directly target LIF/STAT3 to maintain CSCs in an undifferentiated state and maintain the self-renewal of CSCs through the IGF1R signaling pathway. The expression of these key stem cell markers was regulated by feedback loops between these genes and regulated by transcription factors such as GLI and MYC or epigenetic readers like BRD4. JQ1 and SSB1 could synergistically inhibit *GLI* and *MYCN* expression and follow regulated stem cell markers. JQ1 could also reduce pluripotent gene transcription by directly inhibiting BRD4. Therefore, the combination of a BETi (JQ1) and an SMO inhibitor (SSB1) exhibited potential for synergistic blocking of SHH and MYC pathways, which play key roles in regulating the self-renewal and maintenance of MB stem cells, to reduce stem cell proliferation and maintenance and overcome the drawbacks of monotherapy.

The intraspinal metastasis of MB occurs via the cerebrospinal fluid pathway, which is the initial step in ECM degradation. MMPs have been suggested as the key endopeptidases working on degrading ECM components, reducing MB adhesive connections, and enhancing MB metastasis. Within the MMP family, MMP2, as a gelatinase, plays a pivotal role in tumor invasion, and several groups confirm MMP2’s high expression in MB patient tissues. MMP2 secreted and activated by MB cells can degrade collagen IV surrounding tumors and the basement membrane, resulting in the invasion of MB cells into nearby tissues, followed by tumor growth and spread through capillary endothelium and neovascularization. Therefore, inhibiting MMP2 expression and activation could effectively reduce the metastasis of MB. The *MMP2* gene could be directly regulated by BRD4. JQ1 as a BETi could competitively inhibit BRD4 binding on the promoter, reducing *MMP2* mRNA transcription and inhibiting MMP2 expression. Further, the metastasis of MB could also be regulated by GLI1, a key transcription factor in the SHH pathway. The polycomb protein BMI1 is a key regulator of neural stem cells, and BTIC is implicated in the pathogenesis, progression, and metastasis of MB. Previous studies suggested that GLI1 may activate the promoter of *BMI1* and enhance its protein expression [18]. JQ1 and SSB1 both could reduce *GLI* expression, followed by down-regulation of downstream genes *TWIST1* and *BMI1*, indicating the synergistic effects on inhibiting MB metastasis.

Due to the existence of the BBB, passive targeting of NPs is nearly ineffective in transporting them into the brain and uptake by MB cells. To improve the biodistribution of drugs in MB, we used Try-decorated lipid NPs to achieve BBB penetration and MB targeting accumulation. Try-derived NPs could increase cellular uptake based on OCT2 medium cell transport, effectively crossing the BBB and improving their biodistribution. Compared to passive targeting groups, Try-derived NPs significantly increase the drug accumulation of NPs in MB tumor areas with extraordinary treatment efficiency.

Overall, JQ1 and SSB1 combinations exhibit the potential to synergistically inhibit MB progression and metastasis by increasing the cell cycle arrest and apoptosis of MB cells and reducing the frequency of the MB stem cell population. Try-derived NPs, as a promising BBB penetration delivery system, could effectively enhance the intracranial delivery of JQ1 and SSB1 combination and eliminate MB tumors with no observed toxicity.

## Data Availability

Data will be made available on request.
