# Supplementary material for: Tryptamine-Functionalized Lipid Nanocarriers Co-delivering SMO/BRD4 Inhibitors for Synergistic Medulloblastoma Therapy
Source: Biomater Res. 2025 Aug 8;29:0237. doi: 10.34133/bmr.0237 (PMC12332260; doi:10.34133/bmr.0237)
Supplement: Supplementary 1 — Figs. S1 to S11 Table S1 [file bmr.0237.f1.docx]

**Supplementary materials**

**Tryptamine-functionalized lipid nanocarriers co-delivering SMO/BRD4 inhibitors for synergistic medulloblastoma therapy**

Qiyue Wang^1,2*^, Zixu Cui^1^, Chenguang Guo^1^, Yue Zhang^1^, Jinhua Chen^2,3^, Ruitao Zhang^2,3^, Xueming Li^1^, Zhengjie Meng^4*^, Hao Ren^1*^

^1^School of Pharmaceutical Science, Nanjing Tech University, Nanjing 211816, China

^2^ NMPA Key Laboratory for Research and Evaluation of Pharmaceutical Preparations and Excipients, China Pharmaceutical University, Nanjing 210009, China

^3^ Department of Pharmaceutics, School of Pharmacy, China Pharmaceutical University, Nanjing 211198, China

^4^ College of Biotechnology and Pharmaceutical Engineering, Nanjing Tech University, Nanjing 211816, China.

^*^Correspondence: [qiyue.wang@njtech.edu.cn](mailto:qiyue.wang@njtech.edu.cn) (Q. W.); mengjames@njtech.edu.cn (Z. M.); [hren@njtech.edu.cn](mailto:hren@njtech.edu.cn) (H. R.)

**Figure S1**

**
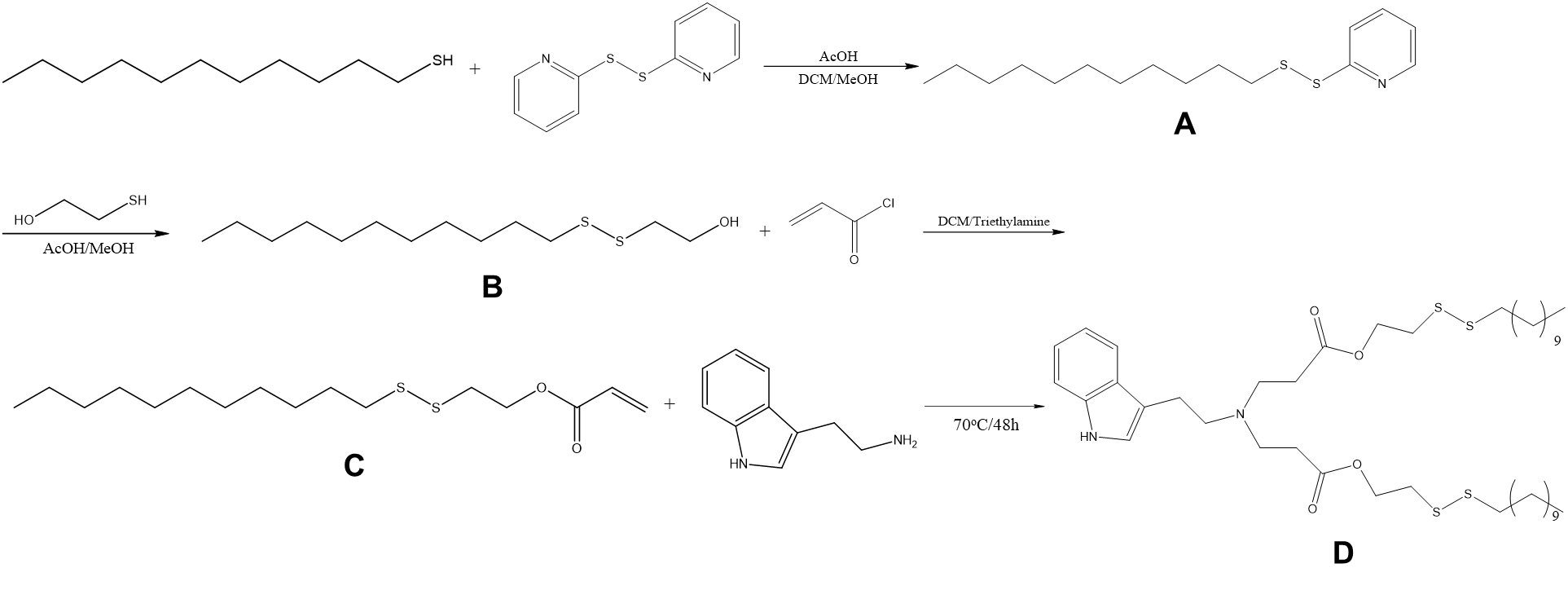
**

**Figure S1.** The synthesis scheme of tryptamine-modified lipids (Try-Lips).

**Figure S2**

**
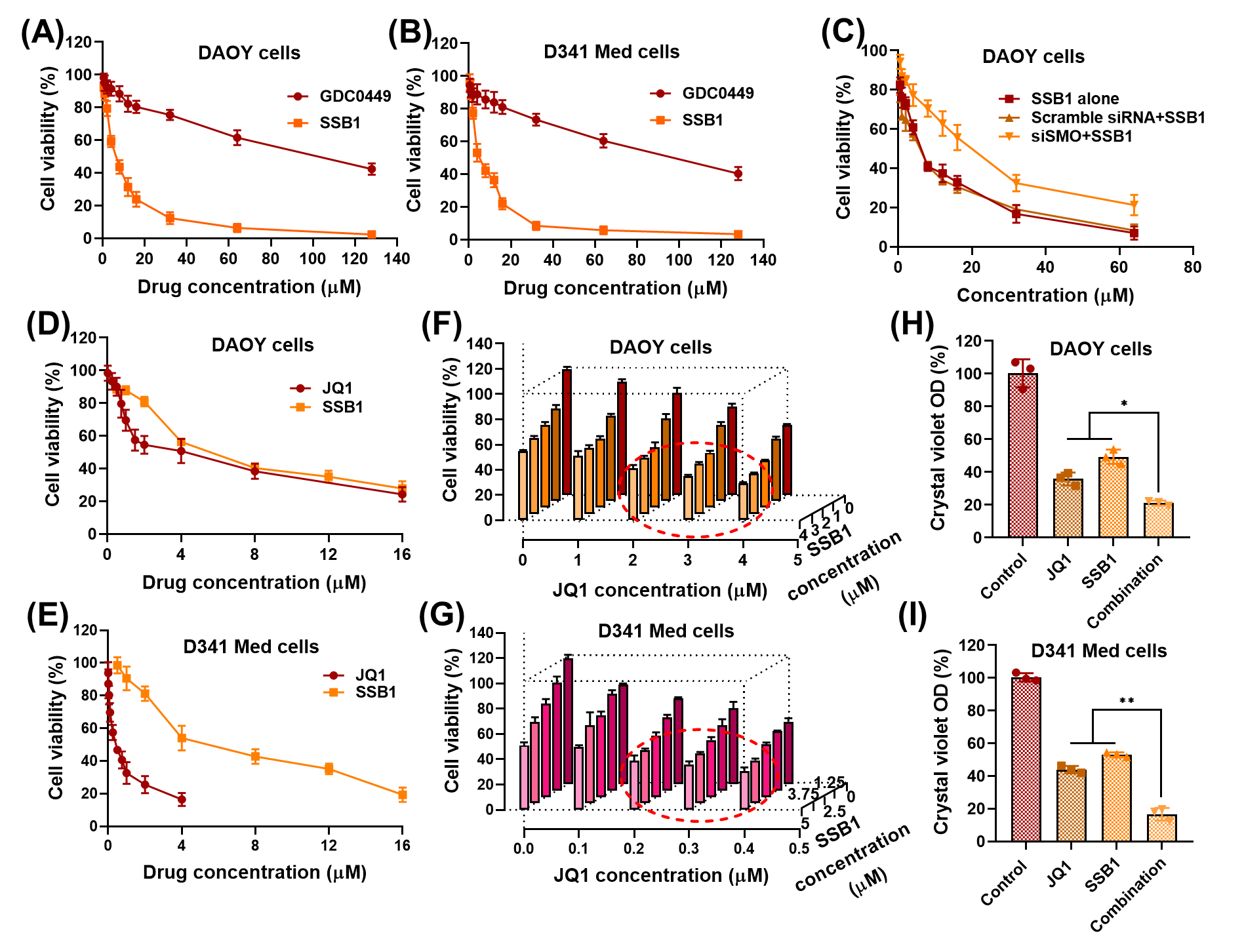
**

**Figure S2.** JQ1 and SSB1 combination efficiently inhibits MB cell proliferation. (A-B) Cell viability of DAOY and D341 Med cells after incubation with GDC0449 or SSB1 for 48 h, respectively. (C) SMO cell viability silenced DAOY cells after 48 h of incubation with SSB1. (D-E) SSB1 and JQ1 inhibit DAOY and D341 Med cell viability in a dose-dependent manner. (F-G) Synergistic cell viability inhibition of SSB1 and JQ1 combination on the DAOY and D341 Med cells. (H-I) Quantifying colony formation assay by measuring crystal violet's optical density (O.D.) in the stained cell colonies after incubation with SSB1 and JQ1 combination.

**Figure S3**

**Figure S3.** The *SMO* mRNA expression in DAOY cells after incubation of different concentrations of lipofectamine/siRNA complexes for 24 h.

**Figure S4**

**Figure S4.** The cell viability of DAOY cells after incubation with JQ1 alone, JQ1 combined with scramble siRNA, and combined with siSMO, respectively.

**Figure S5**

**
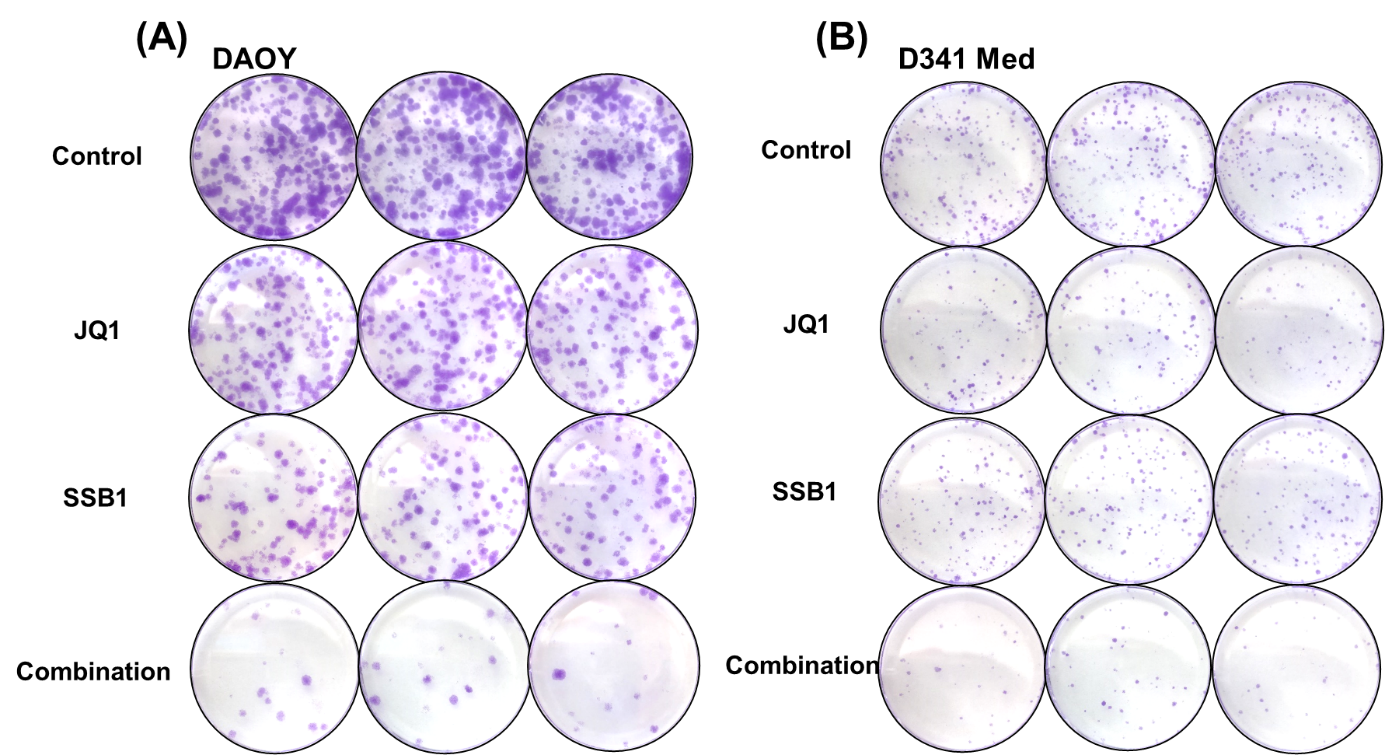
**

**Figure S5.** The colony formation images of (A) DAOY and (B) D341 Med cells after incubation were synergistically inhibited by JQ1 and SSB1 combination at their IC_50_ values.

**Figure S6**

**
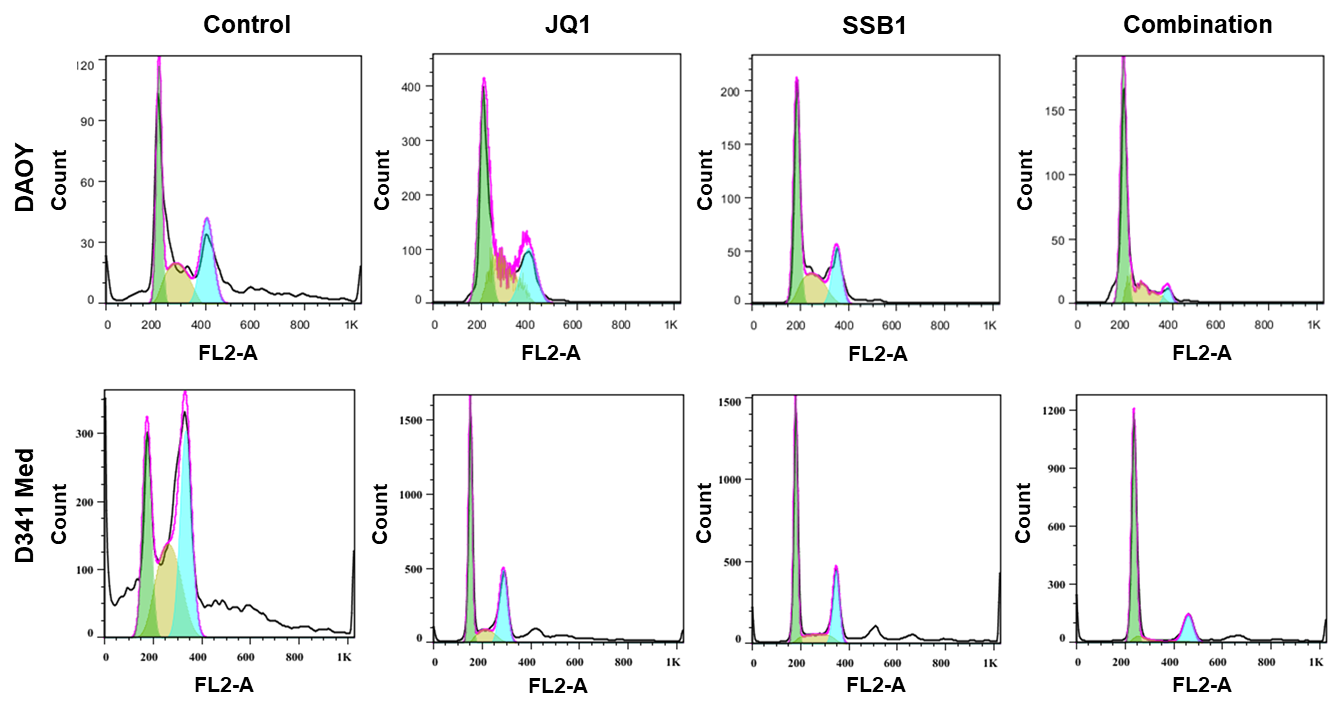
**

**Figure S6.** Representative flow cytometry histograms of JQ1 and SSB1 on cell cycle of DAOY and D341 Med cells. The DNA histograms show the distribution of cell populations in each phase of the cell cycle.

**Figure S7**

**
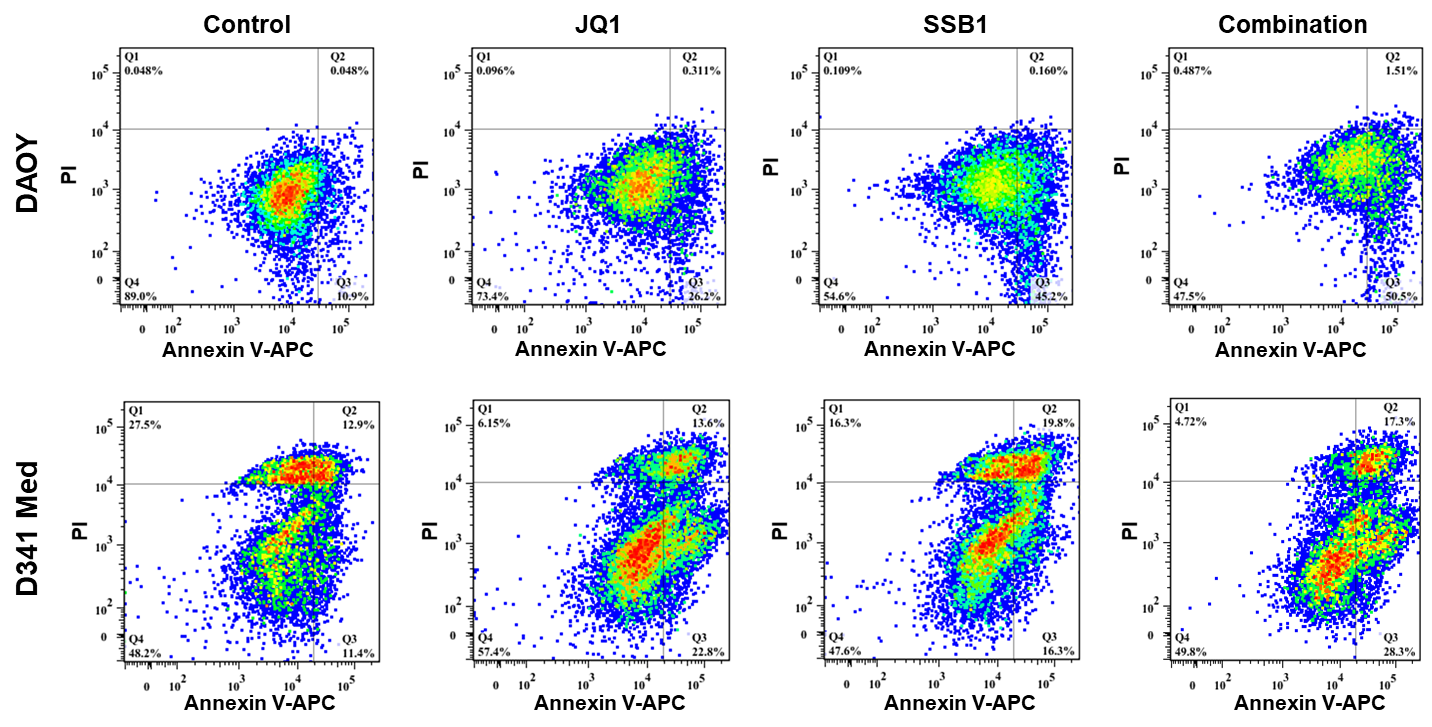
**

**Figure S7.** Representative flow cytometry scatter plots of DAOY and D341 Med cells after incubation of JQ1, SSB1, and their combination, respectively.

**Figure S8**

**Figure S8.** Expression levels of *MMP2* in SVG p12, DAOY, D341 Med, and ONS-76 cells as determined by real-time RT-PCR.

**Figure S9**


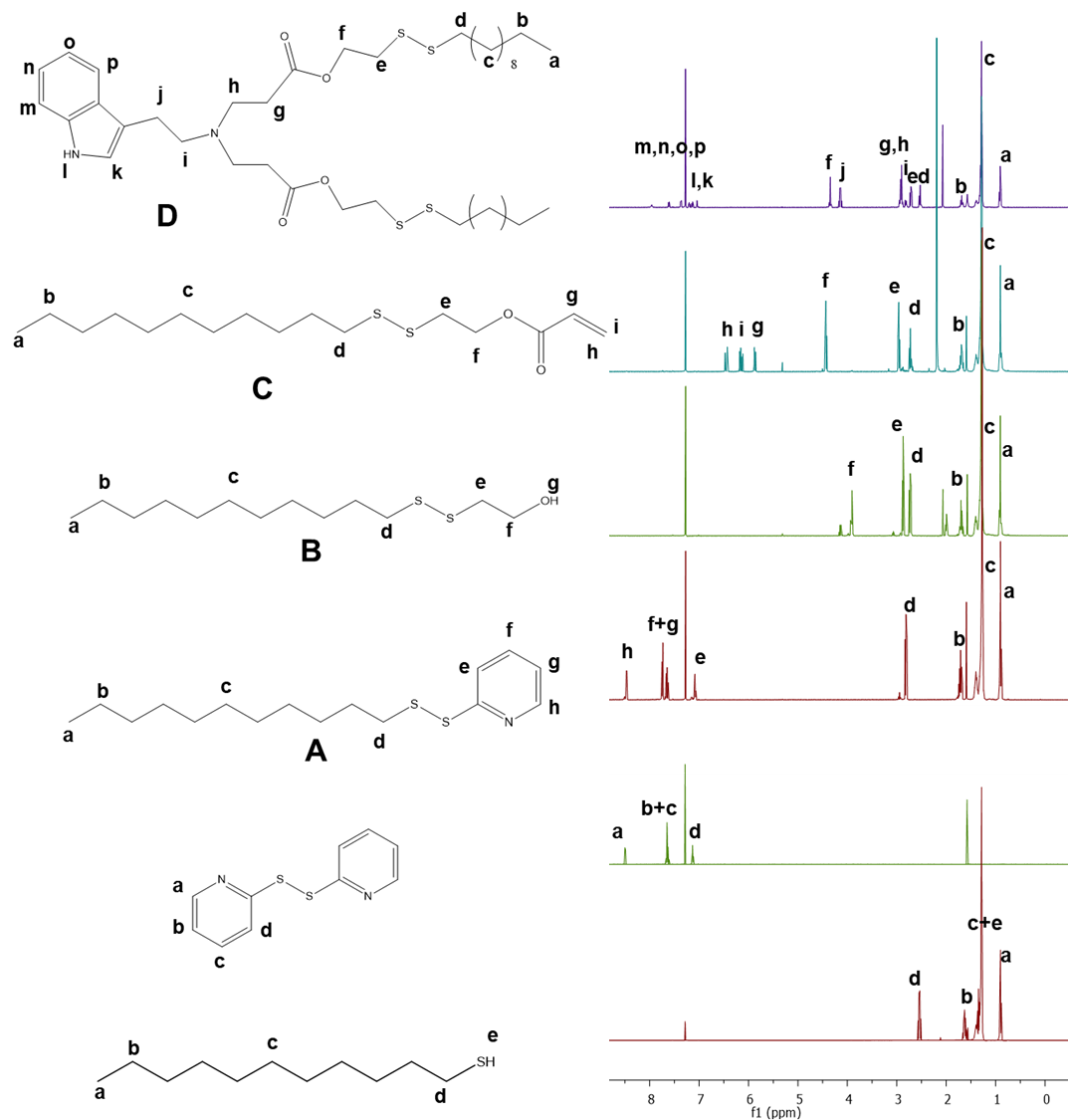


**Figure S9.** The ^1^H NMR of tryptamine-modified lipids (Try-Lips).

**Figure S10**

**
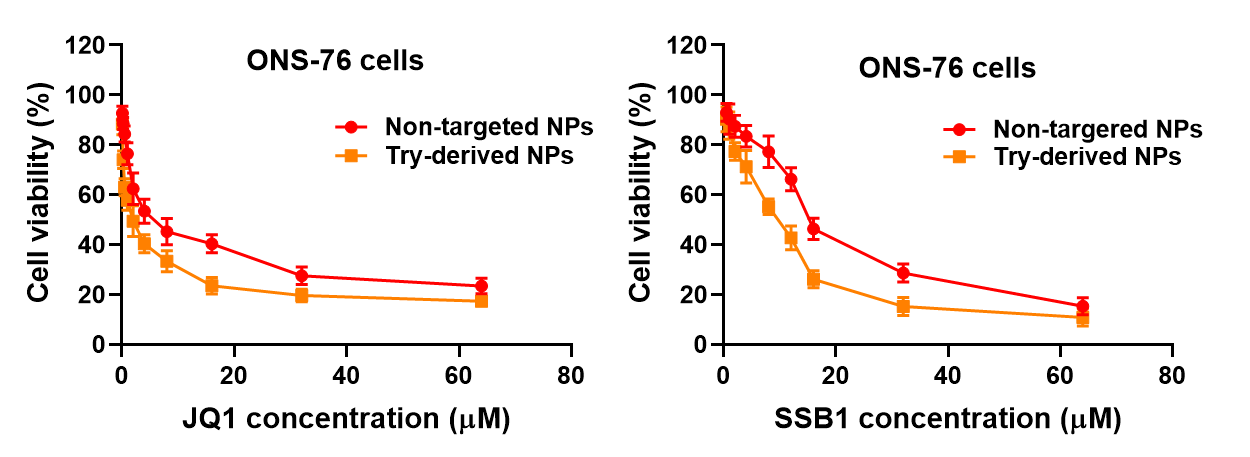
**

**Figure S10.** Cell viability of ONS-76 cells after 48 h incubation with JQ1 and SSB1 loaded non-targeted NPs and Try-derived NPs.

**Figure S11**

**Figure S11.** The changes of TEER value of the in vitro transwell BBB model.

**Table S1. Primers used in the article.**

| Primers Name |  | Sequence |
| --- | --- | --- |
| BAX | Forward | CTGAGCTGACCTTGGAGC |
|  | Reverse | GACTCCAGCCACAAAGATG |
| BRD4 | Forward | ACAACAAGCCTGGAGATGACA |
|  | Reverse | GTTTGGTACCGTGGAAACGC |
| CD133 | Forward | CAACCCTGAACTGAGGCAGC |
|  | Reverse | TTGATAGCCCTGTTGGACCAG |
| CYCLIN D1 | Forward | CAAATGGAGCTGCTCCTGGTG |
|  | Reverse | CTTCGATCTGCTCCTGGCAGG |
| GAPDH | Forward | ACCACAGTCCATGCCATCAC |
|  | Reverse | TCCACCACCCTGTTGCTGTA |
| GLI1 | Forward | CCAACTCCACAGGCATACAGGAT |
|  | Reverse | CACAGATTCAGGCTCACGCTTC |
| GLI2 | Forward | AAGTCACTCAAGGATTCCTGCTCA |
|  | Reverse | GTTTTCCAGGATGGAGCCACTT |
| LAMIN B1 | Forward | CATGAAACGCGCTTGGTAGA |
|  | Reverse | TTGCGCCAGCTTGTACTCATAC |
| MMP2 | Forward | CGCAGTGACGGAAAGATGTGGT |
|  | Reverse | AGAGCTCCTGAATGCCCTTGATGT |
| MYC | Forward | CTGCGACGAGGAGGAGAACT |
|  | Reverse | GGCAGCAGCTCGAATTTCTT |
| MYCN | Forward | CACAAGGCCCTCAGTACCTC |
|  | Reverse | ACCACGTCGATTTCTTCCTC |
| NANOG | Forward | TTTGGAAGCTGCTGGGGAAG |
|  | Reverse | GATGGGAGGAGGGGAGAGGA |
| OCT4 | Forward | CAGTGCCCGAAACCCACAC |
|  | Reverse | GGAGACCCAGCAGCCTCAAA |
| PTCH1 | Forward | TTGCTTGGGAGTCATTAACTG |
|  | Reverse | CCCACAATCAACTCCTCCTGCC |
| SHH | Forward | CCGGCTTCGACTGGGTGTACTA |
|  | Reverse | CGCCACCGAGTTCTCTGCTTT |
| SOX2 | Forward | GGGAAATGGGAGGGGTGCAAAAGAGG |
|  | Reverse | TTGCGTGAGTGTGGATGGGATTGGTG |
